# Proteomic and toxicological analysis of the venom of *Micrurus yatesi* and its neutralization by an antivenom

**DOI:** 10.1016/j.toxcx.2022.100097

**Published:** 2022-02-12

**Authors:** Gianni Mena, Stephanie Chaves-Araya, Johelen Chacón, Enikő Török, Ferenc Török, Fabián Bonilla, Mahmood Sasa, José María Gutiérrez, Bruno Lomonte, Julián Fernández

**Affiliations:** a Instituto Clodomiro Picado, Facultad de Microbiología, Universidad de Costa Rica, San José, Costa Rica; b Department of Human Genetics, Faculty of Medicine, University of Debrecen, 4032, Debrecen, Hungary; c Department of Biophysics and Cell Biology, Faculty of Medicine, University of Debrecen, 4032, Debrecen, Hungary; d Museo de Zoología, Centro de Investigaciones en Biodiversidad y Ecología Tropical, Universidad de Costa Rica, San José, Costa Rica

**Keywords:** Venomics, *Micrurus alleni*, *Micrurus yatesi*, Coralsnake, Antivenom

## Abstract

Coralsnakes belong to the family Elapidae and possess venoms which are lethal to humans and can be grouped based on the predominance of either three finger toxins (3FTxs) or phospholipases A_2_ (PLA_2_s). A proteomic and toxicological analysis of the venom of the coralsnake *Micrurus yatesi* was performed. This species, distributed in southeastern Costa Rica, was formerly considered a subspecies of *M. alleni*. Results showed that this venom is PLA_2_-rich, in contrast with the previously studied venom of *Micrurus alleni*. Toxicological evaluation of the venom, in accordance with proteomic data, revealed that it has a markedly higher *in vitro* PLA_2_ activity upon a synthetic substrate than M. *alleni.* The evaluation of *in vivo* myotoxicity in CD-1 mice using histological evaluation and plasma creatine kinase release also showed that *M. yatesi* venom caused muscle damage. A commercial equine antivenom prepared using the venom of *Micrurus nigrocinctus* displayed a similar recognition of the venoms of *M. yatesi* and *M. nigrocinctus* by enzyme immunoassay*.* This antivenom also immunorecognized the main fractions of the venom of *M. yatesi* and was able to neutralize its lethal effect in a murine model.

## Highlights

• The venom proteome of *Micrurus yatesi* was determined.
• The venom of *Micrurus yatesi* is a Phospholipase A_2_-rich venom.
• When injected in mice, the venom of *Micrurus yatesi* caused muscle damage.
• An antivenom immunorecognized the main fractions of *Micrurus yatesi* venom.
• The antivenom was able to neutralize the lethal activity of the venom of *Micrurus yatesi.*

## Graphical abstract

**Figure:**
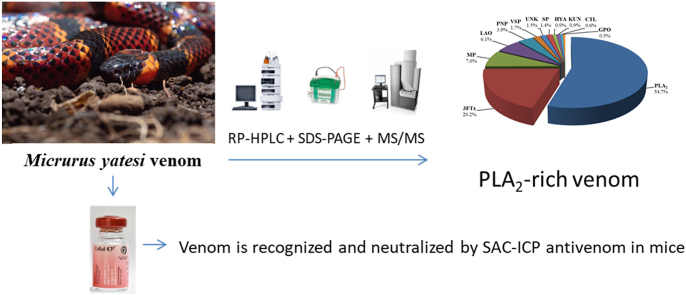

## 1 Introduction

Coralsnakes, genus *Micrurus*, are small to moderate-sized (from less than 50 cm–150 cm in total length) slender elapid snakes that populate diverse habitats, which include lowland rainforests, deserts, and highland cloud forests from southern United States to central Argentina (Campbell and Lamar, 2004; Roze, 1996). Most coralsnakes have a color pattern of some combination of red, yellow or white, and black rings (Campbell and Lamar, 2004). Although a comprehensive phylogenetic analysis of the species that make up the genus is still pending (Zaher et al., 2021), some monophyletic groups have been identified based on the structure of their hemipenes and molecular characters: the group of monadal black ring coralsnakes, which have slender and strongly bifurcated hemipenes; the group of triad pattern coralsnakes with short, bilobed hemipenes; and the group of bicolored coralsnakes with strongly bilobed and slender hemipenes (Slowinski, 1995). Envenomings by coralsnakes are characterized by paresthesia, local pain, palpebral ptosis, dizziness, blurred vision, weakness, slight local edema, erythema, dysphagia, dyspnea, myalgia, salivation and respiratory failure which may lead to death (Bucaretchi et al., 2016). However, regardless of the toxicity of their venoms, bites by these elapids are far less frequent than those caused by pitvipers, representing less than 2% of snakebite envenomations reported in the Western Hemisphere (Bucaretchi et al., 2016).

Transcriptomic and proteomic analyses have revealed that venoms from coralsnakes are characterized by a predominance of phospholipases A_2_ (PLA_2_s) and three-finger toxins (3FTxs), with lower quantities of proteins from other families (Aird et al., 2017; Corrêa-Netto et al., 2011; Lomonte et al., 2016, 2021; Sanz et al., 2019b). Postsynaptically active 3FTxs in these venoms block nicotinic cholinergic receptors by competing with acetylcholine (Moreira et al., 2010), while presynaptically active PLA_2_s hydrolyze phospholipids at the nerve terminal and impair the release of acetylcholine (Dal Belo et al., 2005). From the proteomic point of view, *Micrurus* venoms belong to two main groups depending on the predominant components, i.e., 3FTx-rich venoms and PLA_2_-rich venoms (Fernández et al., 2015; Lomonte et al., 2016, 2021).

Although only a fraction of the total number of coralsnake species has been examined, the expression of these types of venoms might reflect the group's evolutionary history (Lomonte et al., 2016). The selective pressure that mediated this expression pattern is unknown, nor is it clear whether the appearance of PLA_2_-rich venoms has occurred as many independent events within the history of coralsnakes (Lomonte et al., 2021). Integrating more species into the review of venom proteomic profiles, including closely related species, could help elucidate these questions.

*Micrurus alleni* is a widely distributed species found from eastern Honduras to northwestern Panama (Campbell and Lamar, 2004; Roze, 1996). This is a terrestrial and primarily nocturnal snake that inhabits swamps, the vicinity of creeks and rivers, and is found often under leaf litter in primary and secondary forests (Solórzano, 2004).

*Micrurus alleni* was first described as a subspecies of *Micrurus nigrocinctus* by Schmidt (*M. n. alleni*) from specimens collected in Caribbean Nicaragua (Schmidt, 1936). A related form was soon after described by Dunn (1942) as *M. n. yatesi*, honoring Thomas Yates, who collected several specimens in Chiriquí, Panama. The status of these two forms and their distinction from *M. nigrocinctus* was quickly recognized by Taylor (1951) and further supported by Roze (1967) in his early revision of the genus. From these works, *M. alleni* was considered as a nominal species with at least two distinct allopatric populations: *M. a. alleni*, distributed from the Honduran Mosquitia to Caribbean Panama, and *M. a. yatesi*, distributed in the humid forests of the Central and South Pacific of Costa Rica and Chiriquí Province in Panama (Campbell and Lamar, 2004; Roze, 1996).

Although the distinction between these populations is not contested, the use of trinomial nomenclature to distinguish them has not always been accepted (Campbell and Lamar, 2004; Savage and Vial, 1974) and their taxonomic status is currently under review. Previous authors have suggested recognizing *M. yatesi* as a full species separated from *M. alleni* based on distinctive external characters (Campbell and Lamar, 2004; Solórzano, 2004) and divergences in molecular characters (Sasa and Smith, 2001). We follow this recommendation here.

The venom of *M. alleni* from the Caribbean versant of Costa Rica has been previously studied (Fernández et al., 2015) and showed a predominance of 3FTxs. The antivenom used to treat coralsnake envenomings in Central America, prepared against the venom of *M. nigrocinctus* (a phospholipase A_2_-predominant venom)*,* was able to neutralize the lethality of *M. alleni*, albeit with a weaker potency (venom/antivenom proportion of 50 μg/mL to protect all mice) compared to the homologous venom (*M. nigrocinctus,* 300 μg/mL ratio to protect all mice) (Fernández et al., 2015). On the other hand, only few aspects from the venom of *M. yatesi* have been previously studied. An intravenous (i.v.) median lethal dose (LD_50_) of 12.0 ± 2.8 μg/mouse (0.7 ± 0.16 μg/g) and an intraperitoneal (i.p.) LD_50_ of 12.0 ± 1.8 μg/mouse (0.7 ± 0.11 μg/g) were reported by Bolaños (1972). Neurotoxic and phospholipase A_2_ (PLA_2_) activities were also described previously in *M. yatesi* venom (Rosso et al., 1996).

The aim of this work is to report the proteomic composition and toxicological characteristics of the venom of *M. yatesi*, as well as the immunological recognition and neutralization by the antivenom used in Central America to treat coralsnake envenomings.

## 2 Materials and methods

### 2.1 Venoms and antivenom

*Micrurus yatesi* specimens were collected in the South Pacific region of Costa Rica and kept at the Laboratory for Dangerous Animals Research (LIAP) at Instituto Clodomiro Picado, Universidad de Costa Rica.

Venom was obtained from one adult specimen of *M. yatesi* (LIAP 001, 1 km north Wilson Botanical Garden, Copal, San Vito de Coto Brus, Puntarenas Province). The venom was manually extracted, lyophilized and stored at −20 °C until analysis. This venom was used for all experiments, while a small amount of venom from two other individuals (LIAP 094 Hacienda Barú, Bahia Ballena, Osa, Puntarenas Province; LIAP 704 Sabalito, Coto Brus, Puntarenas Province) was used to compare individual variation using RP-HPLC. Venoms from *M. nigrocinctus* (San José, San José Province) and *M. alleni* (Guayacán, Siquirres, Limón Province), consisting of pools from several individuals, were included in some of the assays for comparative purposes. For immunological studies, two batches of a commercial equine antivenom raised against *M. nigrocinctus* produced by Instituto Clodomiro Picado, University of Costa Rica (SAC-ICP) was used: Batch 561, expiry date: July 2018, for ELISA assays; and batch 604, expiry date: May 2021, for neutralization assays. Experiments were performed before the expiry date of the antivenom.

### 2.2 RP-HPLC and SDS-PAGE

Two mg of *M. yatesi* venom were dissolved in 200 μL of solution A (0.1% trifluoroacetic acid; TFA), centrifuged at 15,000×*g* for 5 min to remove debris and separated on a C18 column (250 × 4.6 mm, particle size: 5 μm; Teknokroma) using an Agilent 1200 chromatograph with 215 nm monitoring. Elution was performed with a 1 mL/min flow by applying a gradient of solution A (0.1% TFA) to solution B (0.1% TFA in acetronitrile) as follows: 0% B for 5 min, 0–15% B over 10 min, 15–45% B over 60 min, 45–70% B over 10 min and 70% B over 9 min. The venom fractions were collected manually and dried in a vacuum centrifuge (SpeedVac, Thermo). The fractions were redissolved in water, separated by SDS-PAGE in pre-cast 4–20% gels (Sigma–TruPage™) under reducing conditions and later stained with LabSafe GEL Blue™. The protein bands were cut from the gels and subjected to reduction (10 mM dithiothreitol), alkylation (50 mM iodacetamide) and an *in-gel* digestion with sequencing grade bovine trypsin overnight (in 25 mM ammonium bicarbonate) using an automated digestor (DigestPro MSi, Intavis). Resulting peptides were extracted with a solution of 0.1% TFA and 60% acetonitrile, and concentrated for mass spectrometry analysis.

To evaluate individual venom variation, venoms from 2 other specimens of *M. yatesi* (LIAP 094, LIAP 704) were also analyzed by RP-HPLC using the same conditions. The RP-HPLC profile of the venom of *M. alleni* was also obtained for comparison purposes.

### 2.3 MALDI-TOF/TOF and ESI-MS

Tryptic peptides were mixed with an equal volume of a saturated α-cyano-4-hydroxycinnamic acid matrix (α-CHCA; in 50% acetonitrile, 0.1% TFA). One μL of the mix was spotted onto Opti-TOF 384 plates and dried to later be analyzed in positive reflector mode using a Proteomics Analyzer 4800-Plus instrument (Sciex, Washington D.C., USA). Spectra were acquired using a laser intensity of 3000 and 500 shots/spectrum, using CalMix 5 (ABSciex) as external standards spotted on the same plate. Up to 10 precursor ions were chosen from each MS spectrum for automated collision-induced dissociation MS/MS spectra acquisition at 2 kV, in positive mode (500 shots/spectrum, laser intensity 3500). Resulting spectra were searched using the Paragon® algorithm of ProteinPilot v.4 software (Sciex) against the UniProt/SwissProt database for Serpentes, at a confidence level of ≥95%, for the assignment of proteins to known families. A few peptides with lower confidence scores were manually searched using BLAST (http://blast.ncbi.nlm.nih.gov), and their sequence was confirmed by manual interpretation of MS/MS spectra.

The monoisotopic mass of proteins from prominent RP-HPLC fractions was determined by direct infusion of the fractions (flow rate 5 μL/min), dissolved in 50% acetonitrile and 0.1% formic acid, into a Q-Exactive Plus® mass spectrometer (Thermo Fisher Scientific, USA). MS spectra were acquired in positive mode, using 3.9 kV spray voltage, full MS scan range from 800 to 2500 m/z, and an AGC target of 3 × 10^6^).

### 2.4 Venom protein family abundance

The relative abundance of each venom protein family was estimated by integration of the RP-HPLC peak signals at 215 nm, using Chem Station B.04.01 (Agilent, Santa Clara, California, USA). Densitometry was used for assigning percentual distribution for peak signals with two or more SDS-PAGE bands using Image Lab v.2.0 software (Bio-Rad, Hercules, California, USA).

### 2.5 *In vitro* venom activities

#### 2.5.1 Phopholipase A_2_ activity

Different amounts of *M. yatesi*, *M. alleni* or *M. nigrocinctus* venoms (from 625 ng to 40 μg), dissolved in 25 μL of water, were added to 200 μL of buffer (10 mM Tris-HCl, 10 mM CaCl_2_, 0.1 M NaCl, pH 8.0) in microplate wells. Next, 25 μL of the synthetic substrate 4-nitro-3-octanoyloxy-benzoic acid (4-NOBA, 1 mg/mL in acetonitrile) were added (Holzer and Mackessy, 1996). After a 60 min incubation at 37 °C, absorbance was determined at 405 nm by a microplate reader (Thermo). One unit of PLA_2_ activity was defined as the change of 1 in absorbance. The assay was performed in triplicates.

#### 2.5.2 Enzyme-linked immunosorbent assay (ELISA)

An ELISA was used to assess the ability of the anticoral antivenom produced at ICP to cross-recognize whole *M. yatesi* venom or its RP-HPLC fractions. *M. yatesi*, *M. alleni* and *M. nigrocinctus* venoms were dissolved in sodium phosphate buffer (PBS: 0.12 M NaCl, 0.04 M sodium phosphate, pH 7.2) and adsorbed onto a 96-well microplate (1 μg/100 μL/well) overnight at room temperature. After discarding the excess venom samples, wells were blocked by incubation with 100 μL of PBS that contained 3% bovine serum albumin (BSA) during 30 min. The microplates were washed five times with PBS. A volume of 100 μL of various dilutions (from 1:500 to 1:32000) of antivenom (in PBS with 3% BSA) were added to the microplates, which were incubated during 1 h at room temperature. As a negative control, a mock antivenom prepared from plasma from non-immunized horses was run in parallel under identical conditions on wells with *M. yatesi* venom. After washing the microplates five times with PBS, the antibodies bound to the venoms were detected by the addition of anti-horse IgG/alkaline phosphatase conjugate (Sigma; 1:4000 dilution in PBS with 3% BSA) for 1 h at room temperature, followed by five washes with PBS and the development of final color using *p*-nitrophenylphosphate (1 mg/mL in 0.1 M glycine, pH 10.4, with 1 mM MgCl_2_ and 1 mM ZnCl_2_). The absorbances were registered at 405 nm by a Multiskan microplate reader (Thermo Scientific). All samples were processed in triplicate wells. A similar procedure was used to evaluate the recognition of the most abundant fractions obtained by RP-HPLC (fractions 12, 14, 15, 17, 18, 20, 21, 22, 23, and 26), using instead 0.4 μg/100 μL/well of each fraction and an antivenom dilution of 1:1000, in PBS-BSA for the binding step of equine antibodies.

### 2.6 *In vivo* venom activities

Animal experiments were performed following protocols approved by the Institutional Committee for the Care and Use of Laboratory Animals of the University of Costa Rica (CICUA permit 021-17), using CD-1 mice of either sex, bred at Instituto Clodomiro Picado.

#### 2.6.1 Venom lethality

Various amounts of *M. yatesi* venom (from 3 to 23 μg) dissolved in 100 μL of PBS were injected intravenously (caudal vein) in groups of four mice (body weight between 16 and 18 g). The deaths were recorded after 24 h and the median lethal dose (LD_50_) was calculated by Probit analysis using the BioStat 2008 Professional program (Finney, 1971).

#### 2.6.2 Myotoxic activity

Groups of five mice (18–20 g) received an intramuscular injection of either *M. yatesi* or *M. nigrocinctus* venom (5 μg in 50 μL of PBS) in the right gastrocnemius muscle. A control group received an injection of 50 μL of PBS alone. After 3 h, blood samples were obtained from the tail of each mouse into heparinized capillary tubes. After centrifugation, 4 μL of plasma was used to determine the creatine kinase (CK) activity using a UV-kinetic assay (Wiener Lab, Argentina). CK activity was expressed in Units/L.

Myotoxic activity was confirmed by extracting the injected gastrocnemius of mice 24 h after injection, subsequent to their euthanasia by carbon dioxide inhalation. Muscle tissue was fixed with formalin (3.7%) overnight and routinely processed for embedding in paraffin. Sections of 4 μm thickness were cut and stained with hematoxylin-eosin for histological observation.

#### 2.6.3 Neutralization of lethality

The capacity of the SAC-ICP anticoral antivenom produced in ICP to neutralize the lethal activity of *M. yatesi* venom was assessed by injecting intravenously groups of five mice (16–18 g) with 200 μL of a solution that contained 30 μg of *M. yatesi* venom (equivalent to 3 × LD_50_), which was previously mixed and incubated for 30 min at 37 °C with different dilutions of antivenom to obtain the following venom/antivenom ratios: 100, 200 and 400 μg of venom/mL of antivenom. The control group of mice received the same dose of venom but was incubated with PBS instead of antivenom. Deaths were recorded after 24 h and the median effective dose (ED_50_) was determined using Probit analysis (Finney, 1971).

### 2.7 Statistical analyses

The significance of differences between means of two groups was assessed by Student's *t*-test, or between means of three groups by ANOVA with post-hoc Tukey HSD. Differences were considered significant if *p* < 0.05.

## 3 Results

### 3.1 The venom proteome of *M. yatesi*

**Fig. 1:**
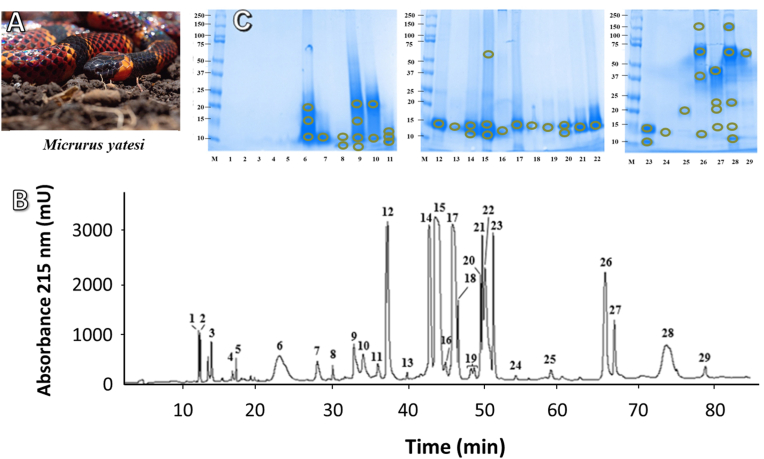
*Micrurus yatesi* specimen (A, photo by Andrés Vega) and elution profile of its venom proteins by RP-HPLC (B), followed by SDS-PAGE analysis (C). A C_18_ column with an acetonitrile gradient was used for venom fractionation, as described in Methods. Further fractionation of proteins was performed using SDS-PAGE under reducing conditions. Molecular weight markers (M) are labeled in kDa. SDS-PAGE bands were excised, *in-gel* digested with trypsin, and analyzed by MALDI-TOF/TOF for protein family assignment, as shown in Table 1.

**Fig. 2:**
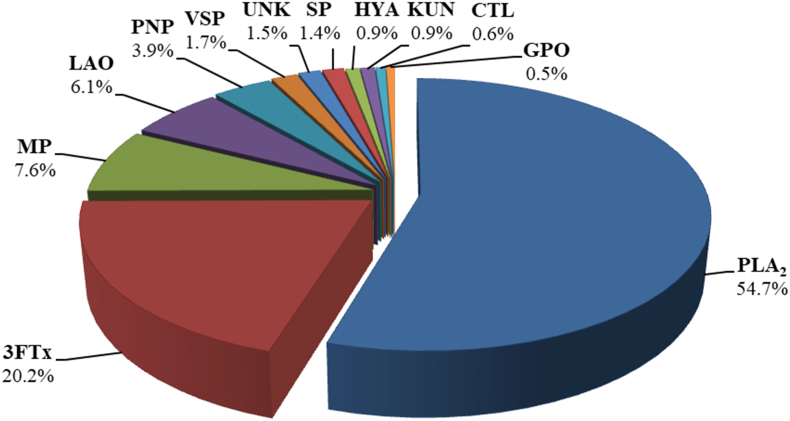
Overall venom composition of *Micrurus yatesi* according to protein families, expressed as percentages of the total protein content. PLA_2_: phospholipase A_2_; 3FTx: three-finger toxin; MP: metalloproteinase; LAO: L-amino acid oxidase; PNP: peptides and/or non-proteinaceous components; VSP: vespryn/ohanin; UNK: unknown/unidentified; SP: serine proteinase; HYA: hyaluronidase; KUN: Kunitz-type serine proteinase inhibitor; CTL: C-type lectin/lectin-like; GPO: Glutathione peroxidase.

**Fig. 3:**
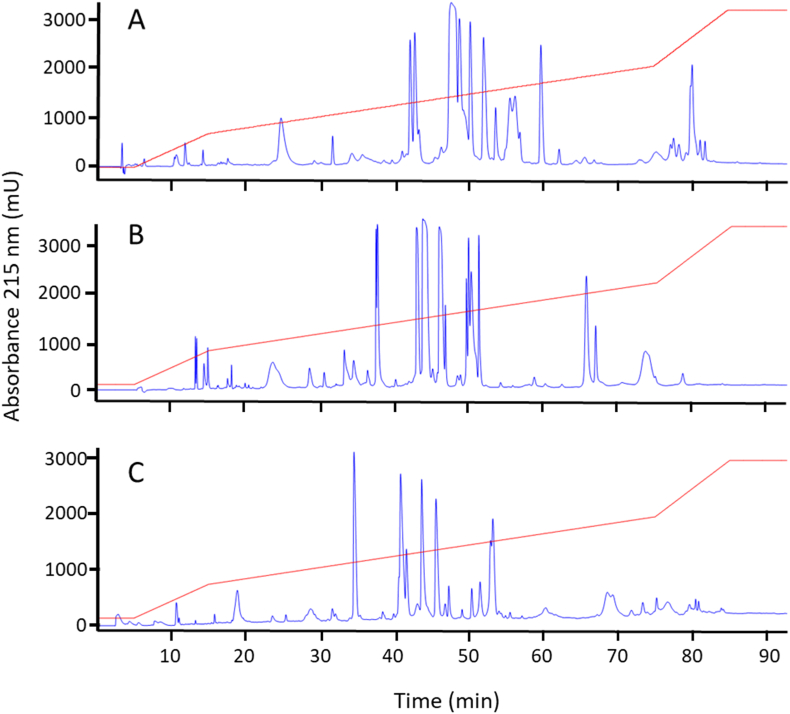
Individual venom variation of *Micrurus yatesi*. Panels A, B, and C show the RP-HPLC profiles of venom from 3 individual specimens of *M. yatesi* under identical conditions. Even though clear differences in the venoms are observed, all 3 venoms show a profile with major fractions in the same time frame. This period (35–55 min) is characterized by the elution of PLA_2_s.

Venom from *Micrurus yatesi* was separated into 29 fractions using RP-HPLC. These fractions were further separated into 48 SDS-PAGE bands (Fig. 1). Most of the SDS-PAGE bands were assigned to a protein family using tandem mass spectrometry. The most abundant proteins in the venom were PLA_2_s (54.7% of total venom proteins, Fig. 2) and 3FTxs (20.2%). Proteins that belong to the metalloproteinase (7.6%), L-amino acid oxidase (6.1%), vespryn/ohanin (1.7%), serine proteinase (1.4%), hyaluronidase (0.9%), Kunitz-type inhibitors (0.9%), C-type lectin/lectin-like (0.6%) and glutathione peroxidase (0.5%) families were detected in lower quantities. Peptidic or non-protein material (peaks 1–5) comprised 3.9% of *Micrurus yatesi* venom. A small percentage (1.5% of the venom proteome) could not be identified.

**Table 1:** Assignment of the RP-HPLC isolated fractions of *Micrurus yatesi* venom to protein families, according to MALDI-TOF/TOF analysis of selected peptide ions from *in-gel* trypsin-digested protein bands.

Peak	%	Mass	Peptide Ion		MS/MS-derived peptide sequence	Conf (%)	Sco	Protein family; related protein
		kDa approx. or (Da)	m/z	z				
1–5	3.9	–	–	–	–	–	–	Peptides or non-proteinaceous components
6a	1.6	18	1283.0	1	BDETXBCCTK	99	10	Three-finger toxin; tr|C6JUP0|C6JUP0_MICCO
			1117.9	1	GCAVTCPBPK	99	11	
			1707.3	1	FSPGXBTSBTCPAGBK	99	20	
6b	2.2	13	1117.9	1	GCAVTCPBPK	99	10	Three-finger toxin; tr|C6JUP0|C6JUP0_MICCO
			1283.0	1	BDETXBCCTK	99	9	
			1707.3	1	FSPGXBTSBTCPAGBK	99	21	
6c	2.2	10	1282.9	1	BDETXBCCTK	99	12	Three-finger toxin; tr|C6JUP0|C6JUP0_MICCO
			1707.2	1	FSPGXBTSBTCPAGBK	99	20	
7	1.5	10	1632.9	1	BFVYGGCGGNANNFK	99	19	Kunitz type serine protease inhibitor; tr|U3FVG9|U3FVG9_MICFL
			1706.9	1	FSPGXBTSBTCPAGBK	99	16	Three-finger toxin; tr|U3FVH8|U3FVH8_MICFL
			1646.9	1	BFXYGGCGGNANNFK	99	14	Kunitz type serine protease inhibitor; tr|R4G317|R4G317_9SAUR
8a	0.3	10	1632.8	1	BFVYGGCGGNANNFK	99	11	Kunitz type serine protease inhibitor; tr|U3FVG9|U3FVG9_MICFL
			1706.9	1	FSPGXBTSBTCPAGBK	99	13	Three-finger toxin; tr|U3FVH8|U3FVH8_MICFL
8b	0.4	8	1375.7	1	ENXCFTMFSAR	90.9	5	Three-finger toxin; tr|C6JUP4|C6JUP4_MICCO
9a	0.8	20	1350.8	1	TXFXVGPSYPEK	99	11	Long chain neurotoxin; tr|U3FYQ0|U3FYQ0_MICFL
			1773.0	1	VCYTXFXVGPSYPEK	99	18	
			997.5	1	FGCAASCPK	99	12	Three-finger toxin; tr|A0A0H4BLZ2|A0A0H4BLZ2_9SAUR
9b	1.0	13	1350.8	1	TXFXVGPSYPEK	99	11	Long chain neurotoxin; tr|U3FYQ0|U3FYQ0_MICFL
			2113.3	1	VCYTXFXVGPSYPEBVXK	99	10	
			2087.2	1	GEBVCYTXFXVGPSYPEK	99	19	
			1773.0	1	VCYTXFXVGPSYPEK	99	19	
			1036.5	1	WGCAASCPK	97.9	9	
9c	0.6	10			Unknown			
9d	0.1	8			Unknown			
10a	2.0	20	1773.0	1	VCYTXFXVGPSYPEK	99	9	Long chain neurotoxin; tr|U3FYQ0|U3FYQ0_MICFL
			1310.7	1	AXEFGCAASCPK	99	20	Three-finger toxin; tr|A0A0H4BLZ2|A0A0H4BLZ2_9SAUR
10b	0.2	10	1310.7	1	AXEFGCAASCPK	99	8	Three-finger toxin; tr|A0A0H4BLZ2|A0A0H4BLZ2_9SAUR
11a	0.3	13	1800.9	1	TTETCADGBNXCFBR	99	10	Three-finger toxin; tr|A0A194APF0|A0A194APF0_9SAUR
			967.6	1	WHMXAPGR	99	10	
			1310.7	1	AXEFGCAASCPK	99	7	Three-finger toxin; tr|A0A0H4BLZ2|A0A0H4BLZ2_9SAUR
11b	0.3	11			Unknown			
11c	0.4	10			Unknown			
12	8.5	15 (13399.9, 13436.9, 13465.8, 13481.9, 13497.9)	1706.0	1	APYN(N^da^)BNFBXDPBR	99	13	Phospholipase A_2_; tr|U3EPD8|U3EPD8_MICFL
			2126.0	1	YGCYCGYGGSGTPVDEXDR	99	14	
			1549.9	1	APYN(N^da^)BNFBXDPK	99	19	
			1373.6	1	CBDFVCNCDR	96.4	8	
13	0.4	15	1096.6	1	APYN(N^da^)BNFK	99	11	Phospholipase A_2;_ tr|U3EPD8|U3EPD8_MICFL
			2126.0	1	YGCYCGYGGSGTPVDEXDR	99	12	
			1549.9	1	APYN(N^da^)BNFBXDPK	99	16	
			1373.6	1	CBDFVCNCDR	99	14	
			1387.6	1	CBEFVCNCDR	96.4	9	Phospholipase A_2_; tr|A0A2H6N4A4|A0A2H6N4A4_MICLE
14a	3.9	16	1373.6	1	CBDFVCNCDR	99	14	Phospholipase A_2_; tr|U3FYP2|U3FYP2_MICFL
			1387.6	1	CBEFVCNCDR	99	13	Phospholipase A_2_; tr|A0A2H6N4A4|A0A2H6N4A4_MICLE
14b	4.0	15 (13260.7, 13276.7, 13291.7)	1373.6	1	CBDFVCNCDR	99	15	Phospholipase A_2_; tr|U3FYP2|U3FYP2_MICFL
			1387.6	1	CBEFVCNCDR	99	13	Phospholipase A_2_; tr|A0A2H6N4A4|A0A2H6N4A4_MICLE
			2855.4	1	SAWDFTNYGCYCGAGGSGTPVDEXDR	99	14	Phospholipase A_2_; tr|A0A2H6MZ62|A0A2H6MZ62_MICLE
15a	0.7	55	1373.6	1	CBDFVCNCDR	99	13	Phospholipase A_2_; tr|U3FYP2|U3FYP2_MICFL
15b	8.3	16	2554.3	1	WTXYSYTCSNGBXTCBDNNTK	99	13	Phospholipase A_2_; tr|A0A289ZBS3|A0A289ZBS3_MICLL
			2236.2	1	CBDFVCNCDRTAAXCFAK	99	19	Phospholipase A_2_; tr|U3FYP2|U3FYP2_MICFL
			1373.7	1	CBDFVCNCDR	99	14	
			1387.7	1	CBEFVCNCDR	99	12	Phospholipase A_2_; tr|A0A2H6N4A4|A0A2H6N4A4_MICLE
15c	7.6	14 (13380.7, 13364.7, 13396.7, 13407.7)	2236.1	1	CBDFVCNCDRTAAXCFAK	99	9	Phospholipase A_2_; tr|U3FYP2|U3FYP2_MICFL
			2857.3	1	PAXDFMNYGCYCGBGGSGTPVDDXDR	99	14	Phospholipase A_2_; tr|Q45Z53|Q45Z53_OXYSU
			2841.3	1	SAWDFTNYGCYCGAGGSGTPVDDXDR	99	14	Phospholipase A_2_; tr|A0A2D4NMC0|A0A2D4NMC0_9SAUR
			2554.3	1	WTXYSYTCSNGBXTCBDNNTK	99	9	Phospholipase A_2_; tr|A0A289ZBS3|A0A289ZBS3_MICLL
			1387.6	1	CBEFVCNCDR	98.9	9	Phospholipase A_2_; tr|A0A2H6N4A4|A0A2H6N4A4_MICLE
16	0.9	13	2678.3	1	CCBVHDBCYDTAEBVHGCWPK	99	9	Phospholipase A_2_; tr|U3FYP2|U3FYP2_MICFL
			1373.7	1	CBDFVCNCDR	99	15	
			1387.7	1	CBEFVCNCDR	94.6	10	Phospholipase A_2_; tr|A0A2H6N4A4|A0A2H6N4A4_MICLE
17	11.4	14 (13229.5, 13258.5)	1373.7	1	CBDFVCNCDR	99	14	Phospholipase A_2_; tr|A0A194AT61|A0A194AT61_9SAUR
			1387.7	1	CBEFVCNCDR	99	11	Phospholipase A_2_; tr|A0A2H6N4A4|A0A2H6N4A4_MICLE
			2678.3	1	CCBVHDBCYDTAEBVHGCWPK	96	9	Phospholipase A_2_; tr|U3FYP3|U3FYP3_MICFL
18	1.7	14 (13195.8)	947.5	1	YHGCWPK	99	10	Phospholipase A_2_; tr|A0A194AS58|A0A194AS58_9SAUR
			1890.0	1	AFVCNCDRTAAXCFGK	99	10	
			2855.4	1	SAWDFTNYGCYCGAGGSGTPVDEXDR	99	19	Phospholipase A_2_; tr|A0A2H6MZ62|A0A2H6MZ62_MICLE
			1329.7	1	CBAFVCNCDR	99	11	
			1041.5	1	AFVCNCDR	99	10	
19	1.2	15	1728.0	1	APYNDBNYNXDXKR	97.7	–	Phospholipase A_2_; AAZ29512.1
20a	1.3	15 (12060.2, 24118.5)	3115.9	1	SPPGBWHBADVTFDSNTAFGSXVVSPDBK	99	20	Vespryn; tr|A0A194AR88|A0A194AR88_9SAUR
			2649.6	1	WHBADVTFDSNTAFGSXVVSPDBK	99	24	
			2198.3	1	ADVTFDSNTAFGSXVVSPDBK	99	30	
			1811.1	1	TVENVGVPBAVSDNPER	99	22	
			1536.0	1	YGTBREWAVGXAGK	99	22	Ohanin; tr|A0A182C6D0|A0A182C6D0_9SAUR
20b	0.8	11 (7512.9)	1306.8	1	XCDVSSXPFXR	99	13	Three-finger toxin; tr|A0A194ATD1|A0A194ATD1_9SAUR
			1462.9	1	RXCDVSSXPFXR	99	16	
			977.7	1	FBWXBBK	99	9	Three-finger toxin; tr|U3EPK7|U3EPK7_MICFL
			1811.1	1	TVENVGVPBAVSDNPER	97.3	7	Vespryn; tr|A0A194AR88|A0A194AR88_9SAUR
21	3.6	(7512.9, 7529.8, 7550.9)	1306.8	1	XCDVSSXPFXR	99	14	Three-finger toxin; tr|A0A194ATD1|A0A194ATD1_9SAUR
			1462.9	1	RXCDVSSXPFXR	99	17	
22	7.1	(7529.8)	1322.8	1	XCDDSSXPFXR	99	16	Three-finger toxin; tr|U3EPK7|U3EPK7_MICFL
			1478.9	1	RXCDDSSXPFXR	99	15	
			1724.0	1	APYNDBNYNXDXBR	99	18	Phospholipase A_2_; tr|U3FYP5|U3FYP5_MICFL
23a	2.6	14 (13646.1, 13684.0)	2527.3	1	CTNDRVWADFVDYGCYCVAR	99	12	Phospholipase A_2_; tr|A0A194AR95|A0A194AR95_9SAUR
23b	0.9	11 (6862.2)	1889.0	1	WYMGTSGDAGCAVTCPR	99	14	Three-finger toxin; tr|U3FYQ9|U3FYQ9_MICFL
24	0.1	13			Unknown			
25	0.6	20	1569.0	1	NVWXGXNDPBBER	99	13	C-type lectin/lectin-like; tr|A0A2H6NF92|A0A2H6NF92_MICLE
			1310.8	1	YTCPXDWXSR	99	15	
26a	0.3	150	1308.6	1	DPDYGMVEPGTK	99	10	Metalloproteinase; tr|U3FWL3|U3FWL3_MICFL
			1054.5	1	TYWHYER	99	11	
26b	3.9	70	1308.6	1	DPDYGMVEPGTK	99	13	Metalloproteinase; tr|U3FWL3|U3FWL3_MICFL
			1054.5	1	TYWHYER	99	11	
			1596.9	1	DRPBCXXNBPXSR	99	14	Metalloproteinase; tr|A0A2D4KKB8|A0A2D4KKB8_9SAUR
26c	1.4	37	1944.9	1	XGVHNVHVHYEDEBXR	99	6	Serine proteinase; tr|A0A2D4Q6K9|A0A2D4Q6K9_MICSU
26d	0.9	14	1260.6	1	DPDYG(M^de^)VEPGTK	99	12	Metalloproteinase; tr|U3FWL3|U3FWL3_MICFL
			1308.6	1	DPDYGMVEPGTK	71.8	6	
27a	2.2	45	2052.9	1	TBPAYBFSSCSVBEHBR	99	9	Metalloproteinase; tr|U3EPC7|U3EPC7_MICFL
			1308.6	1	DPDYGMVEPGTK	99	12	
			1544.8	1	BYXEFYVVVDNR	99	9	
			2114.0	1	XDFNGNTXGLAHXGSXCSPK	99	20	
			1893.0	1	HXNFHXAXTGXEXWTK	99	22	
			1416.7	1	YXEFYVVVDNR	99	15	
27b	0.2	23	1544.8	1	BYXEFYVVVDNR	99	8	Metalloproteinase; tr|A0A194AS47|A0A194AS47_9SAUR
			1184.5	1	DMCFTXNBR	99	10	
			1893.0	1	HXNFHXAXTGXEXWTK	99	17	
			1308.6	1	DPDYGMVEPGTK	99	13	
			2052.9	1	TBPAYBFSSCSVBEHBR	99	8	
			2114.1	1	XDFNGNTXGXAHXGSXCSPK	99	14	
			1416.7	1	YXEFYVVVDNR	99	12	
27c	0.1	20	1263.7	1	SNVAVTXDXFGK	99	9	Metalloproteinase; tr|A0A194AS47|A0A194AS47_9SAUR
			1544.8	1	BYIEFYVVVDNR	99	7	
			1184.5	1	DMCFTXNBR	99	8	
			1893.0	1	HXNFHXAXTGXEXWTK	99	16	
			2114.0	1	XDFNGNTXGXAHXGSXCSPK	99	15	
			1416.7	1	YXEFYVVVDNR	99	12	
27d	0.1	15	1308.6	1	DPDYGMVEPGTK	99	10	Metalloproteinase; tr|U3EPC7|U3EPC7_MICFL
			1893.0	1	HXNFHXAXTGXEXWTK	99	11	
			1222.6	1	VYEMVNXXNK	99	13	
			1544.8	1	BYIEFYVVVDNR	99	14	
			1263.7	1	SNVAVTXDXFGK	99	16	
			1416.7	1	YXEFYVVVDNR	99	14	
			1184.5	1	DMCFTXNQR	97.5	7	
28a	0.9	150	1733.9	1	BDPGXFEYPVBPSEK	99	18	L-amino acid oxidase; tr|A0A2D4G4D6|A0A2D4G4D6_MICCO
			1434.8	1	RXHFBPPXPSDK	99	13	
			2233.1	1	HVVVVGAGMAGXSAAYVXAGAGHK	99	28	
			1310.6	1	RFDEXVGGFDR	99	16	L-amino acid oxidase; tr|A0A194ASA8|A0A194ASA8_9SAUR
			1154.5	1	FDEXVGGFDR	99	15	
			1637.8	1	NDXEGWHVNXGPMR	99	22	
			1963.0	1	TSGDXVXNDXSXXHBXPK	99	24	
			1484.7	1	EADYEEFXEXAR	99	18	
			1833.8	1	EFVBEDENAWYYXK	99	22	
			2275.0	1	XHFAGEYTANDHGWIDSTXK	99	30	
28b	4.6	70	1190.6	1	RRPXGECFR	99	9	L-amino acid oxidase; tr|A0A194ARE6|A0A194ARE6_9SAUR
			1310.6	1	RFDEXVGGFDR	99	19	
			1434.7	1	RXHFBPPXPSDK	99	13	L-amino acid oxidase; tr|A0A2D4G4D6|A0A2D4G4D6_MICCO
			1833.7	1	EFVBEDENAWYYXK	99	24	
			2031.9	1	THRNDXEGWHVNXGPMR	99	27	
			2275.0	1	XHFAGEYTANDHGWXDSTXK	99	32	
			1637.7	1	NDXEGWHVNXGPMR	99	23	
			1484.6	1	EADYEEFXEXAR	99	19	
			3066.4	1	YAMGSXTSFVPYBFBHYFETVAAPVGR	99	14	
28c	0.5	22	1551.8	1	PGGGFV(P^ox^)NFBXFBK	99	15	Glutathione peroxidase; tr|V8P395|V8P395_OPHHA
			1385.7	1	FXVNPBGBPVMR	99	15	
			1944.0	1	HVRPGGGFV(P^ox^)NFBXFBK	99	15	
28d	0.1	15	2235.1	1	HVVVVGAGMAGXSAAYVXAGAGHK	99	20	L-amino acid oxidase; tr|A0A2D4G4D6|A0A2D4G4D6_MICCO
			1833.8	1	EFVBEDENAWYYXK	99	16	
			1637.7	1	NDXEGWHVNXGPMR	99	19	
			1484.7	1	EADYEEFXEXAR	99	19	
28e	0.5	12	1190.6	1	RRPXGECFR	99	11	L-amino acid oxidase; tr|A0A194ARE6|A0A194ARE6_9SAUR
			1034.5	1	RPXGECFR	99	13	
			2235.1	1	HVVVVGAGMAGXSAAYVXAGAGHK	99	44	L-amino acid oxidase; tr|A0A2D4G4D6|A0A2D4G4D6_MICCO
			2251.1	1	HVVVVGAG(M^ox^)AGXSAAYVXAGAGHK	99	20	
			1637.7	1	NDXEGWHVNXGPMR	99	23	
			1484.7	1	EADYEEFXEXAR	99	19	
			2031.9	1	THRNDXEGWHVNXGPMR	99	20	
29	0.9	65	2780.3	1	APMYPNEPFXVFWNAPTTBCBXR	99	14	Hyaluronidase; tr|A0A194APD1|A0A194APD1_9SAUR
			1503.6	1	NFXCBCYBGWK	99	17	
			1810.9	1	DSTAXFPSXYXETXXK	99	20	
			1313.7	1	DYAXPVFVYAR	99	16	
			2031.9	1	BHSDSNAFXHXFPESFR	99	23	
			1544.8	1	EXHPDXSEHAXBR	99	20	
			1903.9	1	HSDSNAFXHXFPESFR	99	25	
			1441.7	1	BDYAXPVFVYAR	99	21	
			1243.6	1	NDBXXWXWR	99	15	

Cysteine residues are carbamidomethylated. X: Leu/Ile; B: Lys/Gln; Confidence (Conf) and Score (Sco) values calculated by the Paragon® algorithm of ProteinPilot®. Mass kDa approx: estimated mass in SDS-PAGE in reducing conditions. Mass values in Da of prominent RP-HPLC peaks were determined by ESI-MS as described in methods, and obtained masses for each RP-HPLC fraction were assigned to sub-fractions according to each SDS-PAGE band mass. Possible, although unconfirmed amino acid modifications suggested by the automated identification software are shown in parentheses, with the following abbreviations: ^da^: deamidated, ^de^dethiomethyl, ^ox^oxidation.

### 3.2 Individual venom variability and comparison with *M. alleni* venom

**Fig. 4:**
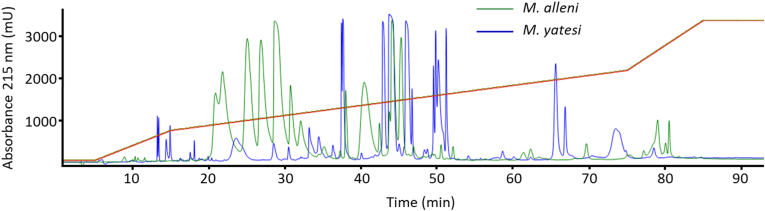
Comparison of the RP-HPLC profiles of venom from one specimen of *Micrurus yatesi* (blue) and venom from *M. alleni* (green). The 3FTx-rich venom of *M. alleni* is characterized by major fractions at the 20–30 min time frame, while the venom of *M. yatesi* contains PLA_2_ fractions that elute at 35–55 min.

Individual venoms of three *M. yatesi* specimens showed clear differences in the RP-HPLC profile, run under identical conditions (Fig. 3). However, prominent fractions of all *M. yatesi* venoms were located in a segment of the chromatogram characterized by the elution of PLA_2_s (35–55 min). The RP-HPLC profile of *M. alleni* venom, a 3FTx-rich venom, showed major fractions at the 20–30 min period (Fig. 4), in contrast with all *M. yatesi* individual venoms.

### 3.3 *In vitro* and *in vivo* activities of *M. yatesi venom*

**Fig. 5:**
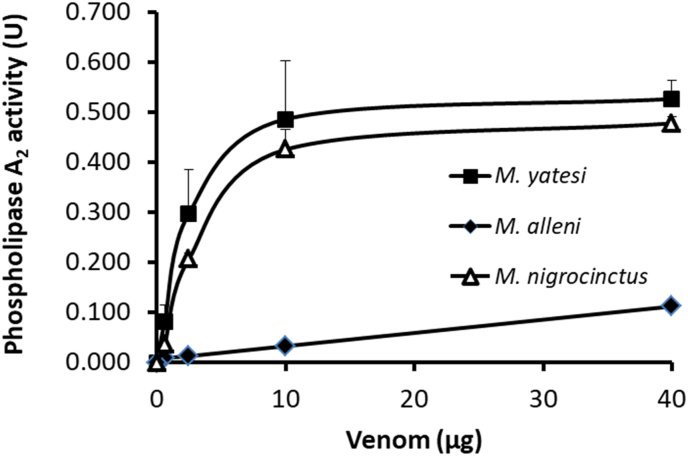
Phospholipase A_2_ activity of the venoms from *M. yatesi*, *M. alleni* and *M. nigrocinctus* on the monodisperse synthetic substrate 4-nitro-3-octanoyloxy-benzoic acid. Different quantities of the venoms were incubated with the substrate for 60 min at 37 °C. One unit is defined as a change of 1 in absorbance at 405 nm. Each point represents mean ± SD of triplicates.

**Fig. 6:**
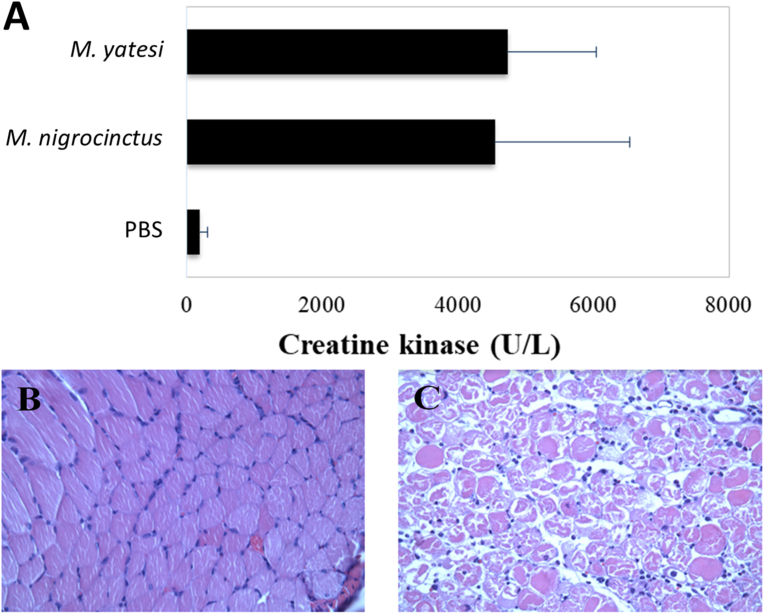
Myotoxic activity caused by the venoms of *Micrurus yatesi* and *M. nigrocinctus* in mice. Animals received an i.m. injection of 5 μg/50 μL of the venoms in the right gastrocnemius and plasma creatine kinase (CK) activity was determined after 3 h (A). The control group was injected with 50 μL of PBS. Bars represent mean ± SD of five mice. The differences between CK activity values of PBS and *M. nigrocinctus* venom, or between PBS and *M. yatesi* venom are significant (p < 0.05). Muscle necrosis was confirmed by histological analyses of muscles injected with *M. yatesi* venom (C), when compared to muscles injected with PBS (B).

Venoms of *M. yatesi* and *M. nigrocinctus* had similar PLA_2_ activities *in vitro* (Fig. 5), which were higher than the activity of the venom of *M. alleni*. The intramuscular injection of *M. yatesi* venom in the gastrocnemius of mice significantly increased plasma CK activity, compared to controls injected only with the vehicle (Fig. 6). The *in vivo* myotoxic activity of *M. yatesi* venom was confirmed by histological analysis of injected muscles which showed widespread distribution of necrotic fibers characterized by hypercontraction and disorganization of the myofibrillar material, as well as edema (Fig. 6). The intravenous (i.v.) LD_50_ of *Micrurus yatesi* venom in mice was 10.1 μg (95% confidence limits: 5.9–14.3 μg) per 16–18 g mouse, or 0.59 μg/g (95% confidence limits: 0.35–0.84 μg/g).

### 3.4 Immunorecognition and neutralization of *M. yatesi* venom by antivenom

**Fig. 7:**
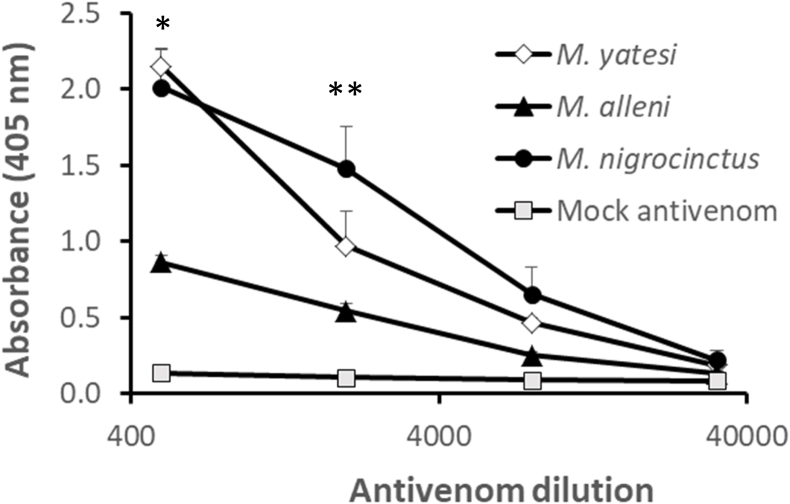
Cross-recognition of *Micrurus yatesi* and *M. alleni* crude venoms by the commercial equine antivenom raised against *Micrurus nigrocinctus* (SAC-ICP), evaluated by ELISA. Venoms were adsorbed to microplates and incubated with various dilutions of antivenom or a mock antivenom prepared using normal horse serum. An anti-horse IgG/alkaline phosphatase conjugate was used to detect bound antibodies, as described in Methods. Each point represents mean ± SD of triplicates. *Differences among all means are statistically significant (p < 0.01) except when the means of *M. yatesi* and *M. nigrocinctus* are compared with each other (no statistical difference). **Differences among all means are statistically significant (p < 0.01 or p < 0.05) except when the means of *M. yatesi* and *M. alleni* are compared with each other or when the means of *M. alleni* and mock antivenom are compared (no statistical difference). Statistical analyses of the other two dilutions are not shown.

**Fig. 8:**
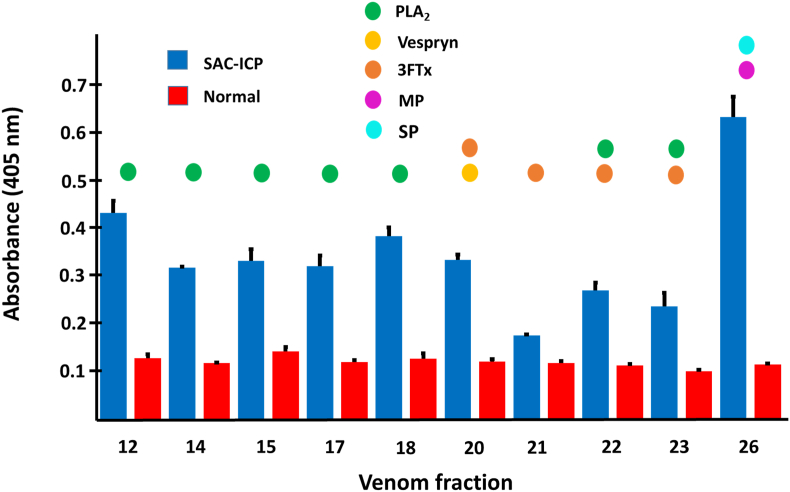
Immunorecognition of major RP-HPLC *Micrurus yatesi* venom fractions by a commercial equine antivenom prepared using *M. nigrocinctus* venom (SAC-ICP). An ELISA assay in which venom fractions were adsorbed onto microplates and bound antivenom antibodies were detected using anti-equine immunoglobulins conjugated to alkaline phosphatase, followed by color development using *p*-nitrophenylphosphate substrate was performed. A mock antivenom was used as a negative control. Each bar represents mean ± SD of triplicate wells. Colored circles above the bars indicate the protein family identified in each chromatographic fraction: Phospholipase A_2_ (PLA_2_), Vespryn/Ohanin, three-finger toxin (3FTx), metalloproteinase (MP), serine proteinase (SP).

The venom of *M. yatesi* was recognized by SAC-ICP antibodies with a similar ELISA signal to the one obtained with *M. nigrocinctus* venom, and a higher signal than the one obtained for *M. alleni* (Fig. 7). The immunorecognition by the antivenom of the most abundant RP-HPLC fractions of the venom was also assessed (Fig. 8). The fraction with the highest signal contained proteins from the metalloproteinase and serine proteinase families, while the least recognized major fraction contained a 3FTx. Fractions that contained PLA_2_s and Vespryn/Ohanin were recognized with a relatively moderate signal by the antivenom. The SAC-ICP antivenom was able to neutralize the lethal activity of *M. yatesi* venom with an ED_50_ of 262 μg of venom/mL of antivenom (95% confidence limits: 187–419 μg/mL).

## 4 Discussion

Several factors including distribution in remote locations, low abundance, venom yield, and limited survival in captivity, have historically precluded a thorough analysis of the venom of *M. yatesi.* Very few specimens of this species have been kept at the serpentarium of ICP along the years. However, the recently collected venom from this species allowed the determination of the proteomic and toxicological characteristics, as well as the immunorecognition and neutralization by an antivenom.

A marked difference in venom composition was observed when the venom of *M. yatesi* was compared with that of *M. alleni.* The former has a predominance of toxins from the PLA_2_ family while the latter has a predominance of toxins from the 3FTx family. This PLA_2_-3FTx dichotomy constitutes a general trend that has been observed in other coralsnake venoms (Fernández et al., 2015; Lomonte et al., 2016, 2021, 2016; Sanz et al., 2016, 2019a). Toxins from both protein families are able to exert neurotoxicity using different mechanisms. The 3FTxs compete with acetylcholine, blocking nicotinic cholinergic receptors at the motor end-plate (Moreira et al., 2010). On the other hand, toxins from the PLA_2_ family impair the release of acetylcholine (Dal Belo et al., 2005) by hydrolyzing phospholipids of the nerve terminal plasma membrane. Differences in other less abundant components were also noted, since Kunitz-type inhibitors and serine proteinases were detected in *M. yatesi* but not in *M. alleni*, while nerve growth factor was reported only in the venom of *M. alleni*. Therefore, the venoms of these closely related species show significant variation in a number of venom protein families.

The toxicological analysis of the venom of *M. yatesi* estimated the intravenous LD_50_ of this venom at 10.1 μg/mouse (0.59 μg/g), whereas the LD_50_ of *M. alleni* was previously estimated in 6.3 μg/mouse (0.37 μg/g) (Fernández et al., 2015). The 95% confidence limits of these determinations overlapped, thus indicating non-significant differences between the toxicity of these venoms. Previously, an i.v. LD_50_ of 12.0 ± 2.8 μg/mouse (0.7 ± 0.16 μg/g) was reported for the venom of *M. yatesi* (Bolaños, 1972). The LD_50_ values of *M. alleni* and *M. yatesi* venoms suggest that they are able to induce lethality in mice through different neurotoxic mechanisms based on the proteomic profiles, i.e., predominantly presynaptically in the case of *M. yatesi* and postsynaptically in the case of *M. alleni*, a hypothesis that deserves further pharmacological studies.

In agreement with proteomic results, the venom of *M. yatesi* displayed significant PLA_2_ activity upon the synthetic substrate 4-NOBA, in a similar fashion as the PLA_2_-rich venom of *M. nigrocinctus.* The 3FTx-rich venom of *M. alleni*, also in agreement with its composition, exhibited very low PLA_2_ activity. When injected in CD-1 mice, the venom of *M. yatesi* exerted muscle damage, evidenced by the increase of plasma CK activity and by histological evaluation of injected muscles. Since *M. yatesi* is a PLA_2_-rich venom, in similarity to *M. nigrocinctus* (Fernández et al., 2011), such myotoxic activity was expected since PLA_2_s are the main myotoxic components in *Micrurus* venoms (Alape-Girón et al., 1999). The 3FTx-rich venom of *M. alleni* has been reported to induce a low (Fernández et al., 2015) to moderate (Gutiérrez et al., 1983) myotoxic effect. Mild myotoxicity has been described in some human cases of envenomings by coral snakes (Bucaretchi et al., 2016), but this effect is not clinically significant. Experimentally, venoms of several species of coralsnakes have been shown to induce prominent myonecrosis in mice (de Roodt et al., 2012; Gutiérrez et al., 1983; Rey-Suárez et al., 2016).

When the immunorecognition of *M. yatesi* venom was evaluated using a commercial equine antivenom, prepared by the immunization of horses with the venom of *M. nigrocinctus*, the PLA_2_-rich venom of *M. yatesi* was recognized to a similar extent as *M. nigrocinctus* venom. In contrast, the 3FTx-rich venom of *M. alleni* was recognized to a lower extent. It has been previously noted that coralsnake venoms with PLA_2_ predominance are better recognized and neutralized by this antivenom than 3FTx predominant venoms (Fernández et al., 2015). Thus, results are in line with the proposal that the compositional 3FTx/PLA_2_ dichotomy of coralsnake venoms is linked with a divergence in their immunological characteristics (Lomonte et al., 2016).

The most abundant fractions of *M. yatesi* venom, which contained mostly toxins from the PLA_2_ family, but also from vespryn, metalloproteinase, serine proteinase, and 3FTx families, were recognized by the SAC-ICP antivenom. The only exception was fraction 21, which contained a 3FTx and displayed a lower signal in the ELISA assay. Larger venom proteins, such as metalloproteinases, are generally better recognized by this coralsnake antivenom than proteins and peptides with a lower molecular mass, such as 3FTxs (Lippa et al., 2019; Rey-Suárez and Lomonte, 2020). This also explains why the fraction that contained a metalloproteinase and a serine proteinase showed the highest signal.

The SAC-ICP antivenom was able to neutralize the lethal activity of the venom of *M. yatesi* in a murine model. This preclinical assay predicts that, in case of envenomings by *M. yatesi*, treatment using this antivenom is likely to be effective. A lower neutralization capacity of the 3FTx predominant *M. alleni* venom was previously described using this antivenom (Fernández et al., 2015). Neutralization assays performed with PLA_2_-rich coralsnake venoms reveal an effective neutralization by this antivenom, while 3FTx-rich venoms are poorly neutralized, or in the case of the venom from *M. mipartitus*, not neutralized (Rey-Suárez et al., 2011).

The close relationship between M. *yatesi* and M. *alleni* has been pointed out in a previous analysis based on mitochondrial DNA (Sasa and Smith, 2001). Although there is scarce knowledge on the natural history of these two coralsnake species, they are likely to share similar ecological niches, and therefore the present findings on their contrasting venom proteomic profiles are intriguing. A handful of stomach records indicate that *M. alleni* often consumes swamp eels (*Synbranchus marmoratus*) and small fossorial colubrids (Solórzano, 2004). Less information is available on the diet of *M. yatesi*, but they have been seen preying on caecilians and small colubrids. Whether these observations reflect differences in the ecological contexts that allowed the selection of uneven venom patterns in different settings is unknown. However, the potential adaptive role of these venom types in immobilizing different prey species deserves further consideration.

## 5 Concluding remarks

The study of venom from *M. yatesi* allowed to determine its venom composition and to compare it with the venom from *M. alleni*. Toxicological analyses, in accordance with the proteomic profile, showed that this PLA_2_-predominant venom possessed significant PLA_2_ activity *in vitro* and caused muscle damage in a murine model. The *Micrurus* antivenom prepared at Instituto Clodomiro Picado recognizes the different fractions of *M. yatesi* venom and neutralizes its lethal activity, hence implying that it is likely to be effective in envenoming by this species.

## Ethical statement

Animal experiments were performed following protocols approved by the Institutional Committee for the Care and Use of Laboratory Animals of the University of Costa Rica (CICUA permit 021-17).

## Declaration of interests

The authors declare the following financial interests/personal relationships which may be considered as potential competing interests: Stephanie Chaves-Araya, Fabián Bonilla, Mahmood Sasa, José María Gutiérrez, Bruno Lomonte and Julián Fernández work at Instituto Clodomiro Picado, where the antivenom used in this study is produced.

## Credit author statement

Gianni Mena: Data curation, Formal analysis, Methodology, writing and editing. Stephanie Chaves-Araya: Investigation and Methodology. Johelen Chacón: Data curation, Formal analysis. Enikő Török: Data curation, Formal analysis, Methodology. Ferenc Török: Data curation, Formal analysis. Fabián Bonilla: Methodology, Resources. Mahmood Sasa: Conceptualization, Methodology, Resources, writing and editing. José María Gutiérrez: Formal analysis, Resources, Funding acquisition, writing and editing. Bruno Lomonte: Conceptualization, Resources, Funding acquisition, writing and editing. Julián Fernández: Conceptualization, Data curation, Formal analysis, Investigation, Methodology, Resources, writing and editing.
